# Simultaneous impregnation and microencapsulation of CaCl_2_ using silica gel and methyl cellulose for thermal energy storage applications

**DOI:** 10.1038/s41598-023-50672-6

**Published:** 2024-03-26

**Authors:** Suboohi Shervani, Curtis Strong, F. Handan Tezel

**Affiliations:** Chemical and Biological Engineering, University of Ottawa, Ottawa, ON Canada

**Keywords:** Energy storage, Chemical engineering

## Abstract

Thermal energy storage utilizing the adsorption of moisture from air is a promising energy storage technology due to its high energy density and minimum heat losses. Salt hydrates and salt hydrate composites, such as calcium chloride (CaCl_2_) and CaCl_2_-based composites, have shown favourable energy storage properties in this area of research. However, these materials have shown issues with stability due to swelling and deliquescence. In this work, CaCl_2_ was stabilized using three methods: impregnation into silica gel, encapsulation in methyl cellulose, and both impregnation and encapsulation stabilization techniques used simultaneously. Therefore, three CaCl_2_-based composites were synthesized. For the first composite, silica gel was impregnated with CaCl_2_. For the second composite, CaCl_2_ was encapsulated by methyl cellulose. For the third composite, silica gel was impregnated with CaCl_2_ and the CaCl_2_ was encapsulated with methyl cellulose. These samples were structurally characterized using scanning electron microscopy as well as Brunauer-Emmett-Teller (BET) to  determine surface area, pore size distribution and nitrogen adsorption isotherms at 77 K. Water vapour adsorption isotherms were also determined at 25 °C for different relative humidities by dynamic vapor sorption (DVS). Similarly, LiCl-based composites were also synthesized and examined in this work, but issues of deliquescence, swelling, and agglomeration made the materials impractical to work with. To determine the prepared materials’ thermal energy storage performance, 2–6 g of each sample was tested in a lab-scale apparatus. This process uses the exothermic adsorption of moisture from ambient air in an open thermal energy storage system. The CaCl_2_ impregnated silica gel that was encapsulated in methyl cellulose showed reasonably high stability and energy storage performance after 3 hydration and dehydration cycles with minimum agglomeration. An energy storage density of 241 kWh/m^3^ (0.87 GJ/m^3^) and specific energy of 630 Wh/kg (2268 kJ/kg) was achieved with this material for 90% inlet relative humidity after a regeneration at 90 °C.

## Introduction

The use of renewable thermal energy sources, like thermal solar power, has been increasing for many years and is expected to continue increasing in the coming decades^1^. Despite the advantages of technologies such as solar thermal, their heat output varies with the amount of solar irradiation^2^. Since solar irradiation varies hourly, daily, and seasonally, it results in an inconsistent thermal power output. Furthermore, this variance in power supply output does not match up with the consumer demand, creating a supply and demand mismatch, with respect to time. This demonstrates the need for and importance of thermal energy storage (TES) technology^3^.

Conventional TES technologies involve sensible and latent heat, but these systems have various disadvantages like low energy storage density, the need for toxic chemicals, and significant heat losses^3^. Adsorption based TES is a newer technology, which in this particular study, involves adsorption of water vapour from air. It exhibits no heat losses during storage over time, high energy densities, and has no need for toxic chemicals. These advantages make it an attractive alternative to sensible and latent heat storage.

Salt hydration/dehydration processes have been attractive options for space heating and domestic hot water applications, due to their high energy density values, their optimal operating temperature ranges and lack of toxic chemicals^3–5^. A popular one involves the hydration/dehydration of CaCl_2_^6–14^. This salt has favourable hydration and dehydration temperatures, is non-toxic, is inexpensive, has a high heat of sorption, and a large water vapour sorption capacity, although it has been criticized for its low temperature lifts^4^. CaCl_2_ and many other hygroscopic salts with high water sorption capacity experience practical issues like deliquescence, swelling and particle agglomeration, which lead to a lack of cyclic stability^4^.

To mitigate these issues researchers have tried impregnating porous matrix materials with CaCl_2_ and other hygroscopic salts^6–14^. A variety of host materials have been used including alumina^15^, carbonaceous materials^16^, cement^17^, porous silica^14^, metal–organic-frameworks (MOFs)^18^ and zeolites^19^. Some researchers have also attempted encapsulation of the salts in polymeric coatings and hollow spheres in order to stabilize them^20–23^. There has also been significant attention related to the encapsulation of phase change materials (PCMs)^24^. These techniques have successfully increased the stability of hygroscopic salts and PCMs, although many of these composites still experience a decrease in performance after multiple hydration and dehydration cycles^25^.

Silica gel is a popular commercial desiccant. It is porous and amorphous, and its pore structure varies depending on the synthesis conditions. It has a high water-vapour sorption capacity, as well as a large surface area and pore volume^26^. As such, many researchers have opted to use this material as a matrix for hydration/dehydration material stabilization. Gordeeva et al. synthesized a silica gel/CaCl_2_ composite and tested it using thermogravimetric analysis (TGA) and differential scanning calorimetry (DSC) analysis. The composite exhibited a sorption capacity of 1.2 g/g and specific energy of 940 Wh/kg at 25 °C and 80% RH (25.4 mbar)^27^. Zhu et al. have also synthesized a silica gel/CaCl_2_ composite which showed a water vapour uptake capacity of 0.73 g/g and specific energy of 264 Wh/kg at 30 °C and 80% RH (34 mbar). This material was tested using a lab-scale energy storage apparatus prototype^25^. Courbon et al. have made improvements to the synthesis method of CaCl_2_/silica gel composites and achieved an energy storage density of 211 kWh/m^3^ (0.76 GJ/m^3^) and a water vapour uptake capacity of 0.4 g/g at a water vapour pressure of 12.5 mbar (30% RH), at an adsorption temperature of 30 °C, and a desorption temperature of 80 °C^14^. There have also been other silica-based materials like MCM-41, SBA-15, and aluminosilicate which have been successfully impregnated with CaCl_2_ and have achieved high energy storage density and water uptake values^8,13,28^. Despite the promising performance of these materials, silica/CaCl_2_ composites lack stability^25,29^. This has prompted the need for new composites and synthesis methods which can adequately stabilize hygroscopic salts like CaCl_2_ and minimize practical issues, like particle agglomeration, while maintaining high energy storage performance.

The micro encapsulation of PCMs has been explored thoroughly in the literature^24^, but the literature about encapsulation of hygroscopic salts is scarce. Shkatulov et al. synthesized hollow mesoporous silica shells and filled them with CaCl_2_, LiCl, and SrBr_2_^21^. The CaCl_2_ material was stable for up to 50 cycles and showed a specific energy of 305 Wh/kg and an energy density of 0.9 GJ/m^3^ (250 kWh/m^3^), with a hydration water vapour pressure of 15 mbar. The LiCl and SrBr_2_ samples were also stable, and they showed energy density values of 0.6 GJ/m^3^ and 0.65 GJ/m^3^ (167 and 181 kWh/m^3^), respectively. Note that the LiCl sample was subjected to 15 mbar of water vapour pressure whereas the SrBr_2_ sample water vapour pressure was 21 mbar. Cuypers et al. reported that encapsulating CaCl_2_ enhanced physical stability and kinetics^22^. Gaeini et al. encapsulated CaCl_2_ with an ethyl cellulose coating; they found that the encapsulated material showed improved cyclic stability and faster kinetics, but a 75% decrease in volumetric energy density of 0.4 GJ/m^3^ (111.1 kWh/m^3^) at 13 mbar hydration water vapour pressure and 20 °C^20^. It was also reported that issues of swelling and agglomeration were still present for the ethyl cellulose coated CaCl_2_. van Ravensteijn et al. encapsulated zeolite 13X and K_2_CO_3_ with various polymer coatings (including ethyl cellulose, polyvinyl alcohol, and hydroxypropyl cellulose) to improve the structural integrity of the thermal energy storage materials^23^. It was found that the permeability of these materials was retained. Similar to ethyl cellulose, methyl cellulose (MC) is a non-toxic and environmentally friendly polymeric material derived from cellulose, often used commercially as an emulsifier or thickener^30^. It has not been previously used for the encapsulation of CaCl_2_, LiCl, or silica gel. This encapsulation technique has also not been used to help stabilize salt-in-matrix composites. However, MC and its derivatives have been used for encapsulation of other materials^31–34^. In the current study, it is used to hold CaCl_2_ in silica gel matrix.

In this study, a novel technology involving the simultaneous impregnation of hygroscopic salts into a porous host matrix and encapsulation by a polymeric coating was implemented. Calcium chloride, a promising hygroscopic salt for thermal energy storage and transformation applications, was stabilized using three methods: impregnation in silica gel, encapsulation in methyl cellulose, and simultaneous impregnation in silica gel and encapsulation in methyl cellulose; the latter two compounds have yet to be seen in the literature. These three materials were synthesized, characterized, and compared to pure silica gel. Additionally, three LiCl composites were synthesized and tested using the same methodologies as for the CaCl_2_-based composites, but the LiCl composites exhibited practical issues, which will be discussed in Section “Energy storage performance”.

## Experimental

### Material preparation

CaCl_2_-based composites have been synthesized via impregnation and encapsulation methods. Silica gel was provided by Xebec Adsorption (Blainville, QC, Canada) and the CaCl_2_, LiCl, and methyl cellulose were purchased from Fisher Scientific (Ottawa, ON, Canada). Methyl Cellulose was purchased through Sigma Aldrich, Canada (Oakville, ON, Canada). A listing of all of the composites and their abbreviated names is provided in Table 1.

**Table 1:** List of all materials tested in this study and their abbreviated names used in this manuscript.

Composite	Acronyms
Pure silica gel	Pure SG
Silica gel/CaCl_2_	SG/CaCl_2_
Methyl cellulose/CaCl_2_	MC/CaCl_2_
Methyl cellulose + silica gel/CaCl_2_	MC + SG/CaCl_2_
Methyl cellulose + silica gel/LiCl	MC + SG/LiCl

**Figure 1:**
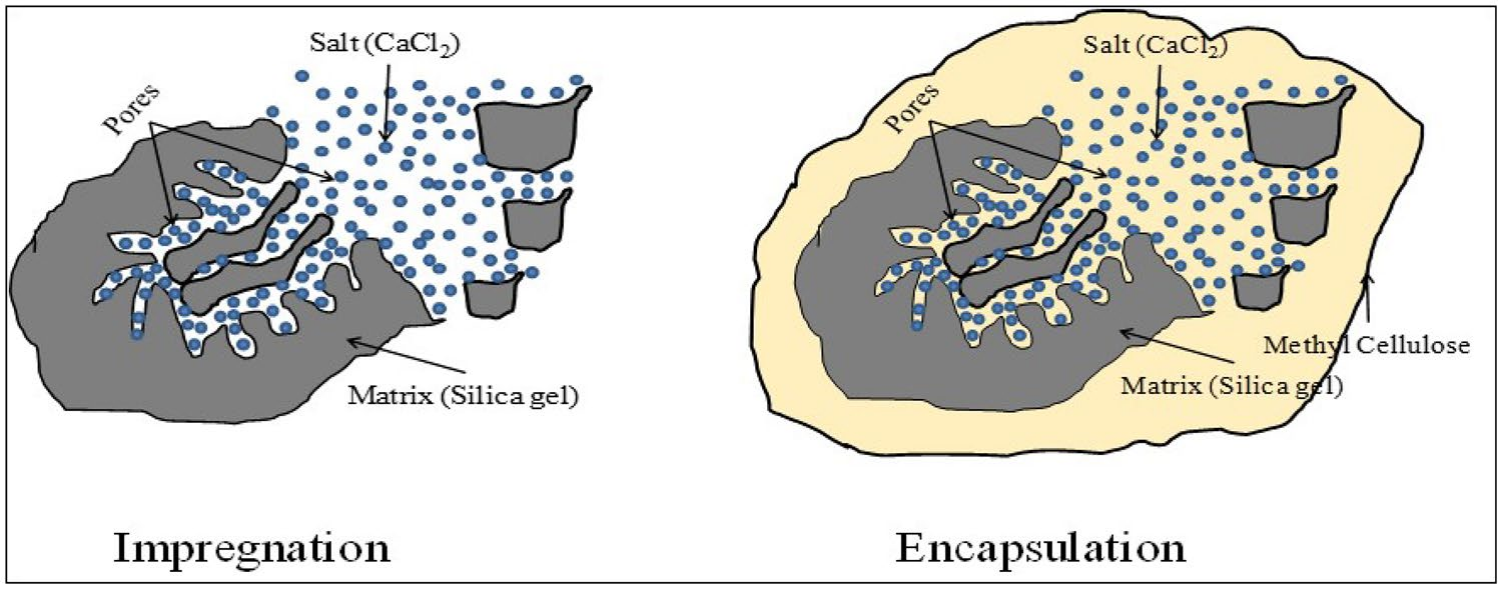
Schematic representation of impregnation and encapsulation mechanism used for the current study.

Figure 1 represents the mechanism used to encapsulate the silica gel and salt hydrate by methyl cellulose. Impregnation is the process where the salt gets onto the surface and into the pores of the silica gel, while encapsulation is the process where a material forms an envelope around the adsorbent. In the current study, encapsulation method is used to hold the CaCl_2_ in the silica gel by methyl cellulose because the deliquescence relative humidity of the CaCl_2_ is very low, and it is not stable in the silica gel without encapsulation after the adsorption of water vapour. The current paper is showing the simultaneous impregnation and encapsulation of salt hydrate into the host matrix for the first time.

#### SG/CaCl_2_ synthesis

First, 30 g of silica gel was kept inside a beaker filled with 100 ml ethanol for half an hour to remove the impurities and contaminations. Silica gel was then filtered from ethanol solution. 15 g of CaCl_2_ and 30 g of silica gel were mixed together in 100 ml de-ionized (DI) water. This thick solution was stirred for 24 h and then was dried in an oven for 6 h at 120 °C.

#### MC/CaCl_2_ synthesis

15 g of CaCl_2_, and 10 g of MC were mixed in 100 ml de-ionized (DI) water and 5 ml ethanol was poured into the mixture. The solution was stirred for 48 h and was dried in an oven for 24 h at 90 °C.

#### MC + SG/CaCl_2_ synthesis

First, 30 g of silica gel was kept inside a beaker filled with 100 ml ethanol for half an hour to remove the impurities and contaminations. Silica gel was then filtered from ethanol solution. 15 g CaCl_2_, 10 g MC and 30 g of silica gel were mixed together in 100 ml de-ionized (DI) water and 5 ml Ethanol was poured into the mixture. This thick solution was stirred for 48 h and then it was dried in an oven for 24 h at 90 °C.

#### MC + SG/LiCl synthesis

First, 30 g of silica gel was kept inside a beaker filled with 100 ml ethanol for half an hour to remove the impurities and contaminations. Silica gel was then filtered from ethanol solution. 15 g LiCl, 10 g MC and 30 g silica gel were mixed in 100 ml de-ionized (DI) water and 5 ml of ethanol was poured into the mixture. This thick solution was stirred for 48 h and then it was dried in an oven for 24 h at 90 °C.

### Structural characterization

Structural characterization of the prepared CaCl_2_ hybrids has been performed by scanning electron microscopy (SEM) by using JSM-7500F field emission scanning electron microscopy (FE-SEM) of JEOL. The porosity, surface area, pore volume have been measured by Brunauer–Emmett–Teller (BET) surface area analyzer (Micromeritics Instrument Corporation) at the McGill University, Montreal, Quebec, Canada.

### Energy storage apparatus and methodology

**Figure 2:**
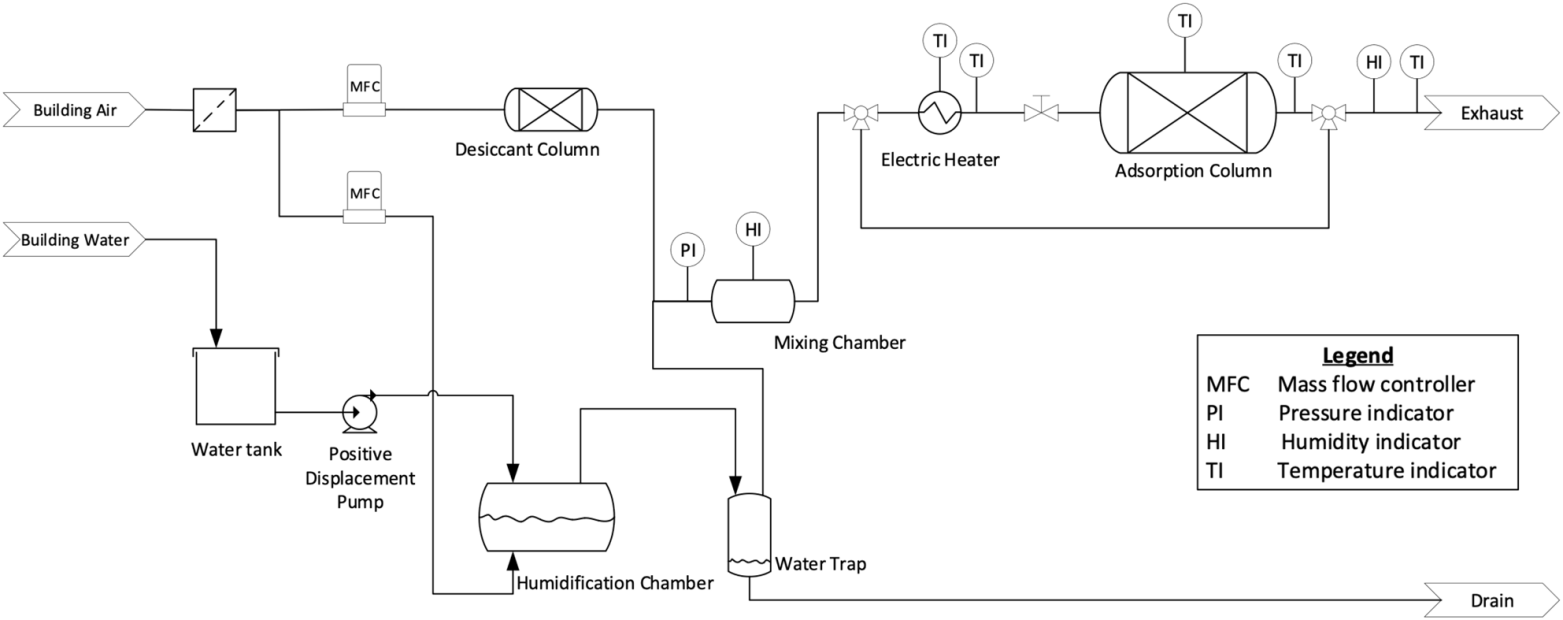
Schematic diagram of the lab-scale energy storage apparatus that was used to test the energy storage performance of the materials prepared in this study. All measurements were recorded electronically using LABVIEW.

A lab scale energy storage apparatus was used to test the energy storage performance of the hybrids prepared. The system and methodology used is similar to those described in Hua, et al.^35^. A schematic diagram of the energy storage apparatus is shown in Fig. 2. The stainless-steel sorption column, covered in fiberglass insulation, has a volume of 7.15 cm^3^, with an inner diameter of 1.09 cm and a length of 7.67 cm. Small pieces of glass wool were placed at the inlet and outlet of the column to avoid particles exiting the column. The column was filled with 2–6 g of the composite adsorbent materials prepared, depending on the bulk density. The composites were crushed and sieved to a 7 × 20 mesh size (0.841–2.83 mm) using a mortar and pestle. Note that the MC/CaCl_2_ sample was rubbery and elastic, so unlike the more brittle samples, it could not be crushed with a mortar and pestle. Therefore, it was chopped into finer pieces using a knife before it was packed into the column.

To dehydrate the sample and store thermal energy, air with an RH of 0–3% at room temperature ($\approx$ 22 °C) was heated to 90 °C and passed through the column at a rate of 12 L per minute (LPM). The dehydration continued until the RH reading at the outlet of the column was less than 3% for at least 15 min. Following dehydration, the column was isolated using valves and left to cool to room temperature overnight. In an actual application of this technology, the heat source would be coming from solar thermal heat or waste heat to be stored.

During hydration, the stored energy was released by humidifying dry building air at room temperature to 50 or 90% RH and allowing the humid air to pass over the column, at a flow rate of 12 LPM. Adsorption of water vapour takes place in the column, which is an exothermic process that generates heat. Since the column is nearly adiabatic, this results in a temperature increase for the dry air leaving the system at the outlet of the column. This temperature change is monitored and recorded by temperature sensors connected to the column as shown in Fig. 2. The energy released during hydration is therefore calculated using Eq. (1), with the information from the column inlet and outlet temperatures, the mass flow reading from the mass flow controller, and the heat capacity of the air at the given temperature and humidity level. Note that zero is the time at the start of the hydration and $t$ is the time during the experiment.

1 $$Q_{hydration} = \mathop \smallint \limits_{0}^{t} \dot{m}_{air} C_{p,air} \left( {T_{out} - T_{in} } \right)dt$$

The maximum thermal power was the product of the maximum temperature difference between the inlet and outlet column temperatures ($\Delta {T}_{{\text{max}}}$), the specific heat capacity of air, and the mass flow rate as shown in Eq. (2):

2 $$\dot{Q}_{max} = \dot{m}_{air} C_{p,air} \Delta T_{max}$$

The water vapour mole fraction, $x_{{{\text{H}}_{{2}} {\text{O}}}}$, and absolute humidity, *H*, were calculated using Eqs. (3) and (4), respectively. Then, based on the difference in inlet and outlet absolute humidity over the course of the water-vapour hydration breakthrough experiment, the water vapour uptake capacity was calculated using Eq. (5). Note that at the inlet of the column, the total pressure (${P}_{tot}$) is assumed to be 101.3 kPa plus the reading on the pressure gauge by the mixing chamber (Fig. 1), and the pressure at the outlet of the column was measured to be 101.3 kPa.

3 $$x_{{{\text{H}}_{2} {\text{O}}}} = \frac{{RH p_{{{\text{H}}_{2} {\text{O}}}}^{sat} }}{{100 P_{tot} }}$$

4 $$H = \frac{{x_{{{\text{H}}_{2} {\text{O}}}} \times M_{{{\text{H}}_{2} {\text{O}}}} }}{{x_{{{\text{H}}_{2} {\text{O}}}} \times M_{{{\text{H}}_{2} {\text{O}}}} + \left( {1 - x_{{{\text{H}}_{2} {\text{O}}}} } \right) \times M_{air} }}$$

5 $$q = \frac{{\left[ {\mathop \smallint \nolimits_{0}^{t} \dot{m}_{air} \times \left( {H_{inlet} - H_{outlet} } \right)dt} \right]}}{Mass of the adsorbent}$$

Based on ${Q}_{hydration}$, the energy storage density (ESD), and specific energy (SE) can be calculated. The ESD was computed by dividing ${Q}_{hydration}$ by the column volume V (7.15 cm^3^) and the SE was calculated by dividing ${Q}_{hydration}$ by the mass of the adsorbent sample in the column using Eqs. (6) and (7), respectively:

6 $$ESD = \frac{{\mathop \smallint \nolimits_{0}^{t} \left( {m Cp \Delta T} \right)dt}}{V}$$

7 $${\text{SE}} = \frac{{ Q_{hydration} }}{Mass\;of\;the\;adsorbent }$$

## Results and discussion

### Scanning electron microscopy (SEM)

**Figure 3:**
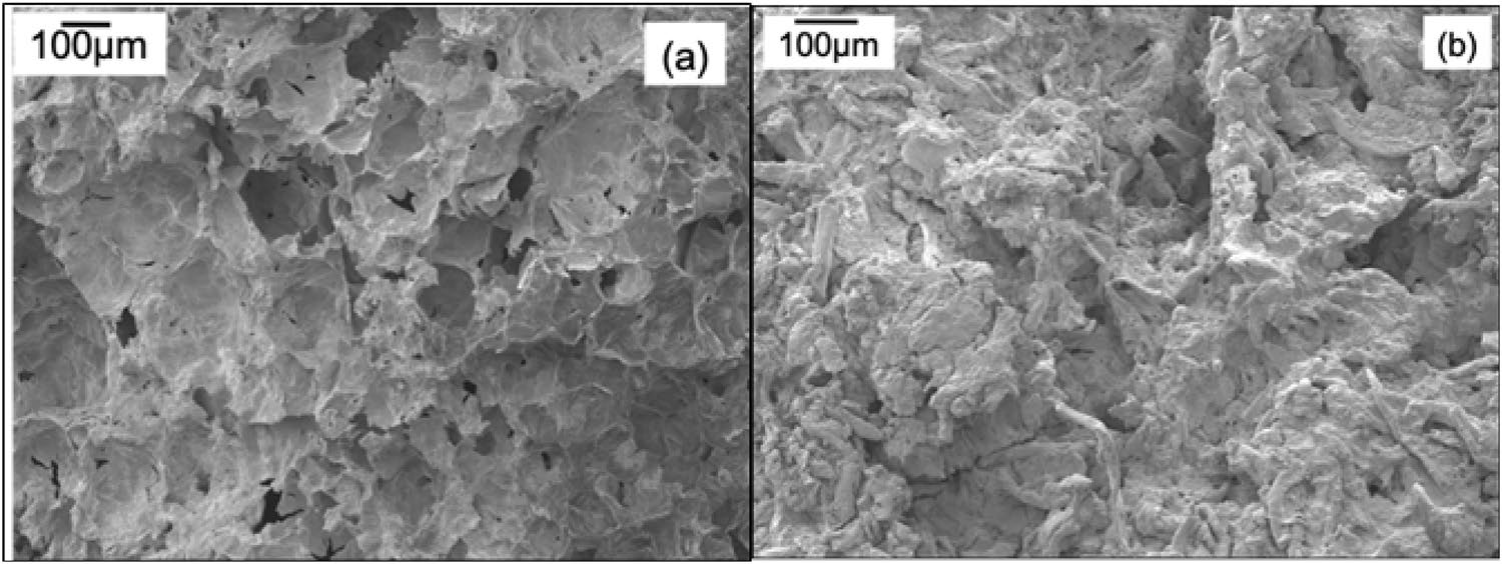
(**a**) Scanning electron microscopy image of MC/SG/CaCl_2_ hybrid. (**b**) Scanning electron microscopy image of MC/CaCl_2_ hybrid.

Figure 3a represents the SEM image of MC/SG/CaCl_2_ hybrid, and Fig. 3b represents the SEM image of MC/CaCl_2_ hybrid. It is clear from these images that with the incorporation of SG, the pore size of the hybrid increases. This is also confirmed with the existence of macropores in the BET isotherm results discussed in Section “BET (Brunauer–Emmett–Teller) measurements”, and more adsorption sites are generated to adsorb the water vapour. Both samples are showing rough and macroporoous structure, which is due to the encapsulation by methyl cellulose.

### BET (Brunauer–Emmett–Teller) measurements

**Figure 4:**
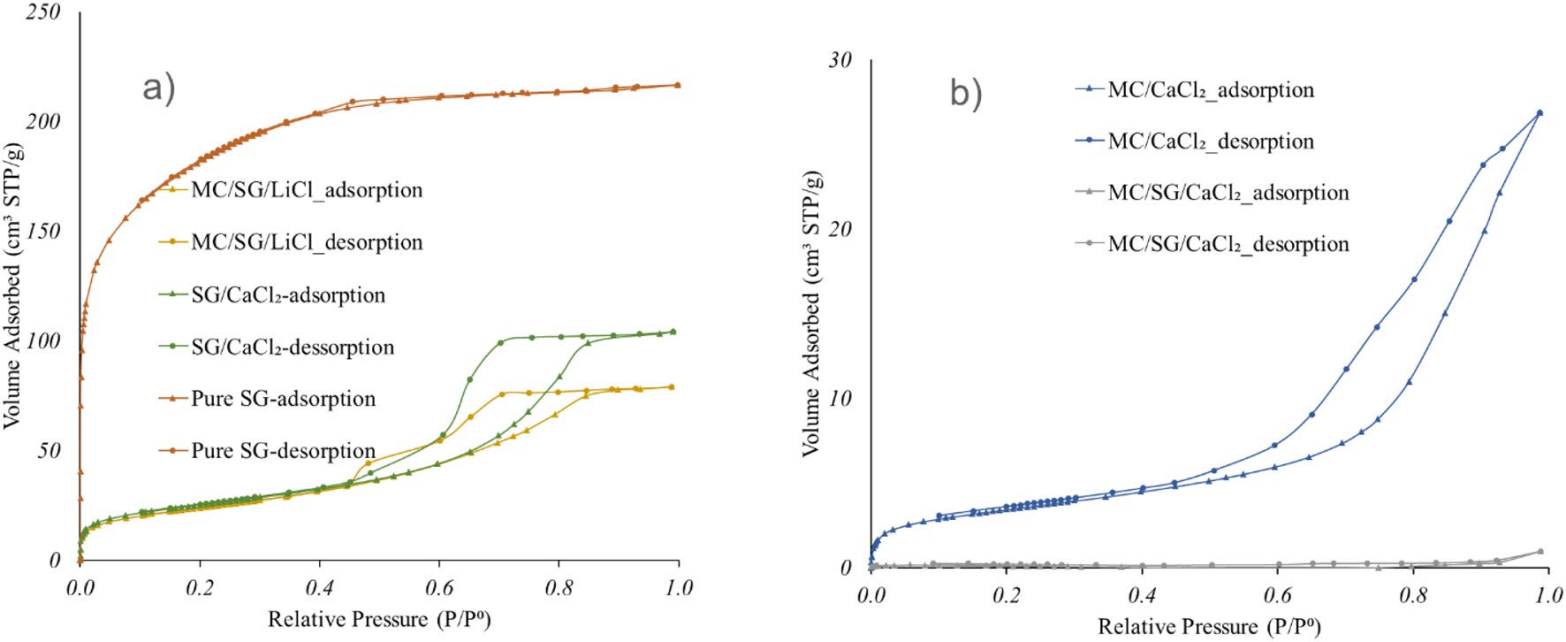
(**a**) Nitrogen adsorption and desorption isotherms of MC/SG/LiCl hybrid, SG/CaCl_2_ and Pure SG at 77K. (**b**) Nitrogen adsorption and desorption isotherms of MC/CaCl_2_ hybrid, and MC/SG/CaCl_2_ hybrid at 77K.

Table 2 shows the results of BET measurements for all five adsorbents reported in this paper. The surface area of pure SG is 689.7 m^2^/g, SG/CaCl_2_ is 89.3 m^2^/g, MC/CaCl_2_ is 12.2, and MC + SG/CaCl_2_ is 0.60. This shows the gradual reduction in the surface area due to incorporation of CaCl_2_ in pores of silica gel which is blocking up the pores. Furthermore the encapsulation of SG + CaCl_2_ by methyl cellulose results in much reduced surface area and non-porous structure. This is also confirmed from the N_2_ isotherm results in Fig. 4a, b. Looking at the nitrogen sorption measurement shown in Fig. 4a, only pure SG is showing the Type-I isotherm. For a Type I isotherm, the amount adsorbed approaches a limiting value. A steep uptake at very low *P*/*P*^0^ is due to enhanced adsorbent-adsorbate interactions in narrow micropores (micropores of molecular dimensions), resulting in micropore filling at very low *P*/*P*^0^.

**Table 2:** Surface area, pore size, and pore volume of different adsorbents used in this study.

Adsorbents	Surface area (m^2^/g)	Pore size (Å)	Pore volume (cm^3^/g)	Type of N_2_ isotherm (See Fig. 4)	Type of hysteresis loop (See Fig. 4)
Pure SG	689.7	30	0.000240	I	-
SG/CaCl_2_	89.3	40–150	0.000080	IV	H2
MC/SG/LiCl	84.5	40–150	0.000179	IV	H2
MC/CaCl_2_	12.2	100–300	0.000012	IV	H3
MC/SG/CaCl_2_	0.6	200	0.000034	IV	H3

**Figure 5:**
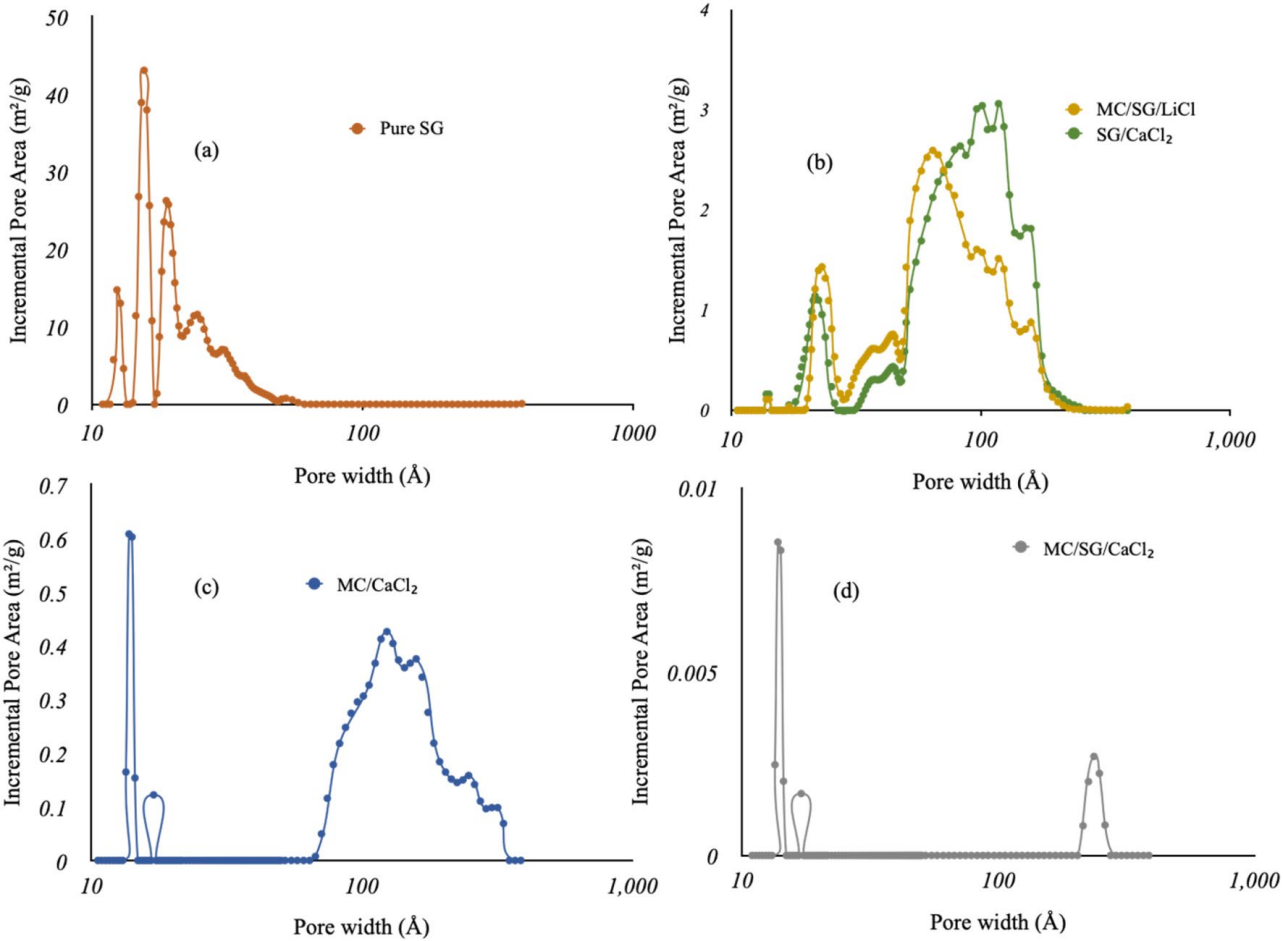
Pore size distribution of (**a**) Pure SG, (**b**) MC/SG/LiCl hybrid, and SG/CaCl_2,_ (**c**) MC/CaCl_2_ hybrid, and (**d**) MC/SG/CaCl_2_ hybrid.

It can also be observed from Table 2 and Fig. 5 that the pore size of silica gel has substantially increased by its impregnation as well as the encapsulation of the CaCl_2_ and LiCl in the silica gel matrix.

Both SG/CaCl_2_ and MC/SG/LiCl composites are showing Type-IV isotherms as can be seen in Fig. 4a. For a Type IV isotherm, capillary condensation at high relative pressures is accompanied by hysteresis between adsorption and desorption branches. This occurs when the pore width exceeds a certain critical width, which is dependent on the adsorption system and temperature. The presence of CaCl_2_ slightly modifies the hysteresis loop, giving H2 kind of hysteresis loop^36^ in SG/CaCl_2_ and MC/SG/LiCl as can be seen in Fig. 4a. H2 hysteresis is associated with complex pore structures in which network effects are important. The steep desorption branch, an important feature of H2 hysteresis, is associated to pore-blocking or percolation in small range of pores or to cavitation-induced evaporation. This confirms the incorporation of salt into the pores of the matrix. The salt completely filled the smallest pores of the matrix and partially filled the largest pores of the matrix. Nevertheless, the apparition in the composite of larger pores than in the matrix is surprising. This could be explained by a different interaction between N_2_ molecules and the surface of composite containing salt which could interfere with the pore size calculation.

Both composites MC/CaCl_2_ and MC/SG/CaCl_2_ (Fig. 4b) show the H3 hysteresis loop. In H3 loop the adsorption branch resembles a Type II isotherm and the lower limit of the desorption branch is normally located at the cavitation-induced P*/*P^0^. Loops of this type are associated with the pore network consists of macropores which are not completely filled with pore condensate^36^.

In comparison to the pure porous matrix (Table 2), a significant decrease in both the specific surface area and the total pore volume of the composite is observed in agreement with the incorporation of the salt into the pores of the silica gel matrix. In comparison to the pure silica gel, decrease of the external surface area is observed, indicating the possible presence of a small amount of salt into the micropores and/or onto the surface of the matrix^37^. This could lead to a problem of stability of the composite. That is why the cycling stability of the composite will be improved in the present work by encapsulation, before a potential use in a storage system.

### Energy storage performance

**Figure 6:**
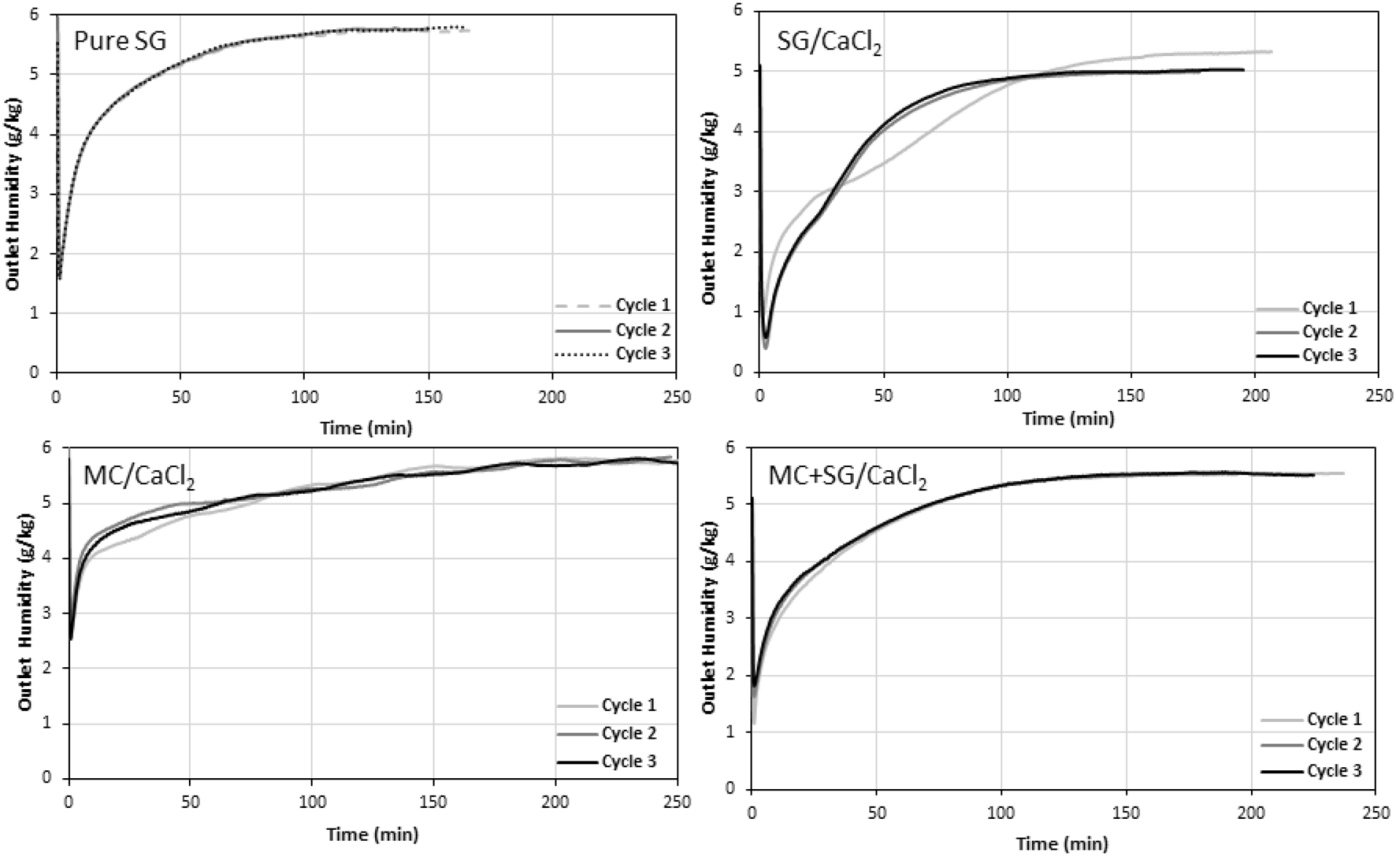
The concentration breakthrough curves for all three hydration cycles for pure silica gel and the three CaCl_2_-based composite materials. The inlet RH was set at 50%, the flow rate was 12 LPM and the regeneration temperature was 90 °C.

**Figure 7:**
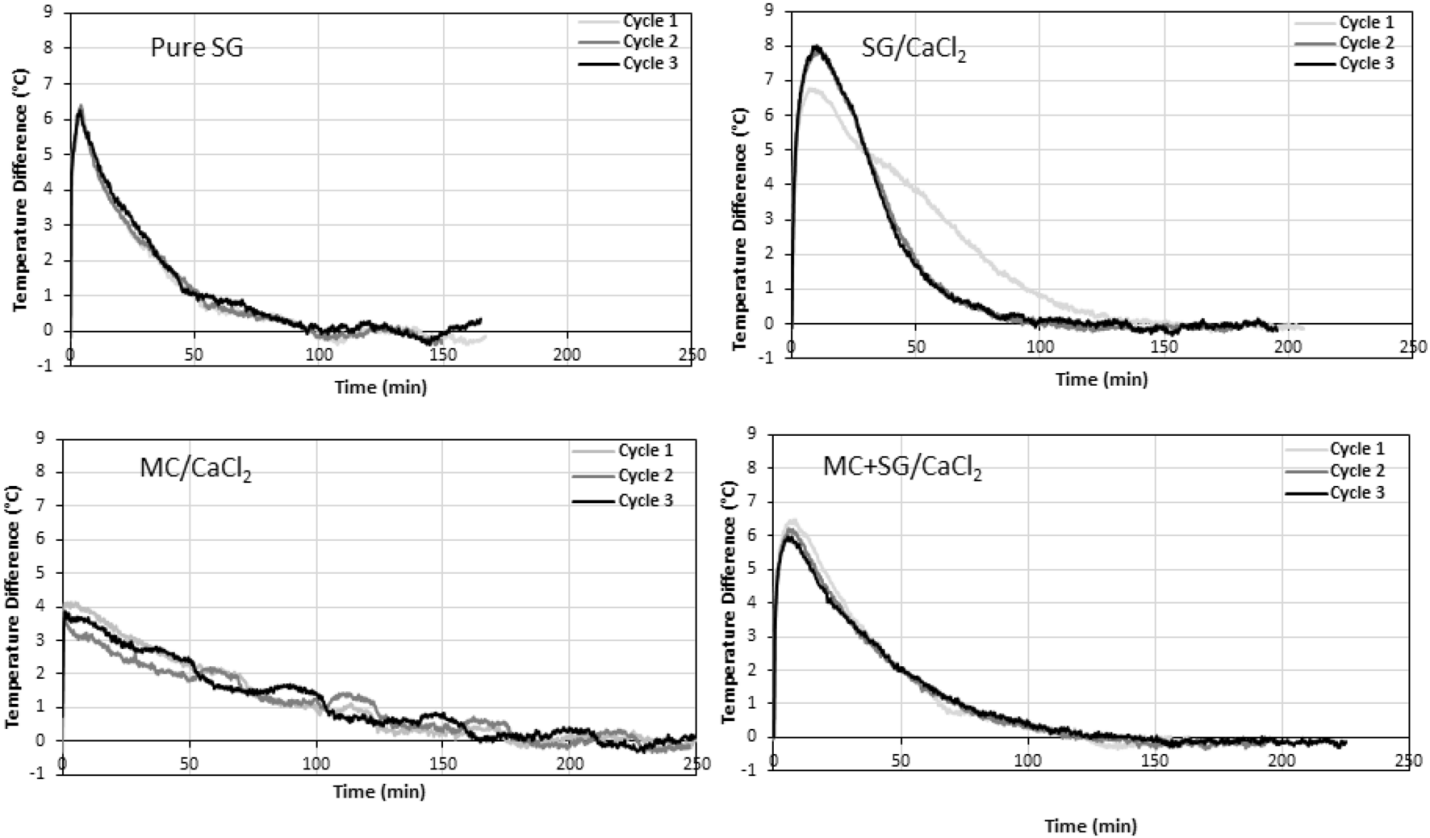
Temperature breakthrough curves for all three hydration cycles for pure silica gel and the three CaCl_2_-based composite materials. The inlet RH was set at 50%, the flow rate was 12 LPM and the regeneration temperature was 90 °C. The temperature difference was defined as the difference between the inlet and outlet temperature of the column.

The concentration and temperature breakthrough curves for each material are plotted for all three cycles for silica gel and the three CaCl_2_-based composites in Figs. 6 and 7, respectively. Note that not all the trials exhibited the same outlet humidity at the end of the hydration. This is because the pressure drop was not the same for each sample and therefore the inlet total pressure was different for each sample. This resulted in variance in the inlet humidity, as per Eqs. (3) and (4).

**Figure 8:**
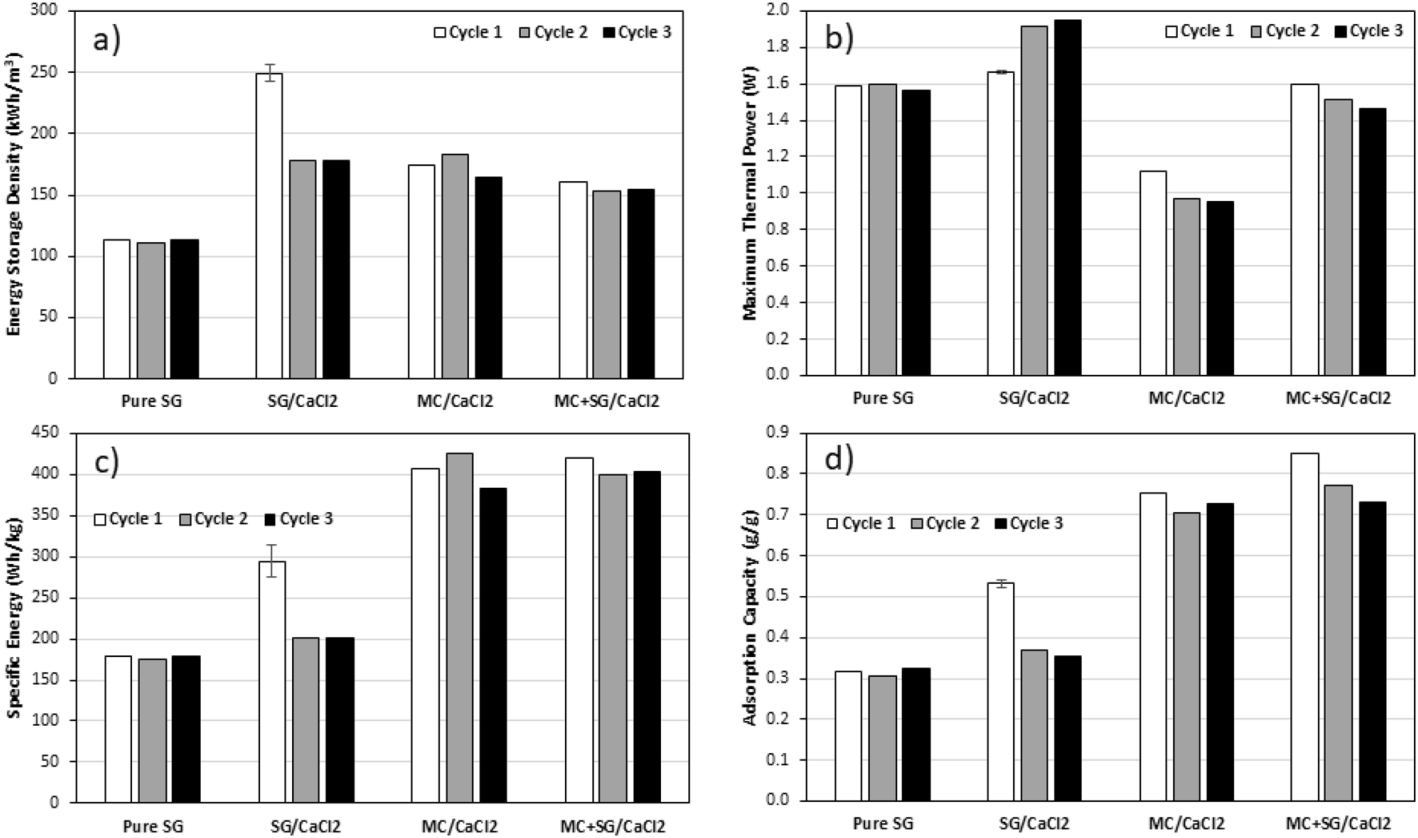
(**a**) Energy storage density, (**b**) Maximum thermal power, (**c**) Specific energy, (**d**) Water-vapour uptake capacity for all samples studied for three hydration/dehydration cycles performed at adsorption inlet relative humidity of 50%, and flow rate of 12 LPM, after regeneration at 90 °C. The error bars on the first cycle of SG/CaCl_2_ are based on repeated experiments with fresh sample.

In Fig. 6, we can see that the pure silica gel breakthrough curves are nearly superimposed for cycles 1–3, showing excellent stability for different cycles. It is also apparent that the SG/CaCl_2_ exhibits a significant change after the first cycle, which completely changes the shape of the concentration breakthrough curve, and makes it resemble more that of pure silica gel. The MC/CaCl_2_ sample showed a more gradual and sluggish concentration breakthrough behavior. It also shows higher variations in humidity readings than the other samples since it was more greatly affected by the atmospheric temperature. This is because the MC/CaCl_2_ sample was known to swell slightly so the column was packed very loosely, meaning that there was less sample to act as a heat sink and therefore it was more directly affected by the ambient temperature. This is further supported by the temperature oscillations seen in Fig. 7 for MC/CaCl_2_. The MC/SG/CaCl_2_ concentration breakthrough curve was like that of pure silica gel but with a slightly lesser slope and there is a slight noticeable increase in the rate of change of the outlet humidity for 2nd and 3rd cycles. This is likely because the water–vapor uptake capacity is decreasing slightly, as seen in Fig. 8d.

In Fig. 7, the pure silica gel temperature breakthrough curve shows a sudden and sharp increase at the beginning of the experiment, followed by a very long tail. All three cycles exhibit similar behavior. For the first cycle for the SG/CaCl_2_ sample a large initial temperature lift is observed, then the temperature difference decreases almost linearly and finally tails off. However, in the subsequent two cycles, the maximum temperature lift is higher, but the decrease and tailing is more sudden, ultimately reducing the area under the curve and therefore the ESD. However, the second and third cycles for SG/CaCl_2_ are almost coincident, further supporting that there is a significant change in the material properties after the first cycle but very little change after the second. The MC/CaCl_2_ temperature breakthrough shows oscillations due to changes in ambient temperature, as mentioned before. This is attributed to the fact that the MC/CaCl_2_ experienced slight swelling and was therefore packed very lightly into the column, leaving large void space and increasing the relative effects of the ambient temperature since there is less sorbent material to act as a heat sink. Finally, the MC + SG/CaCl_2_ sample shows an initial peak in temperature difference then a long tail, similar to pure silica gel but with a broader tailing. As the cycle number increases, the maximum temperature lift decreases slightly, which causes a decrease in maximum thermal power that was observed in Fig. 8.

All three composite materials and pure silica gel were tested at an adsorption inlet relative humidity of 50% at room temperature ($\approx$ 22 °C) after a regeneration temperature of 90 °C to compare their performances as thermal energy storage materials. The flow rate during both hydration and dehydration was 12 LPM. Each material underwent three consecutive dehydration and hydration cycles. Based on these experiments, the ESD, maximum thermal power, SE, and water vapour uptake capacity were calculated for each of these three cycles. The results for all these experiments are shown in Fig. 8.

**Figure 9:**
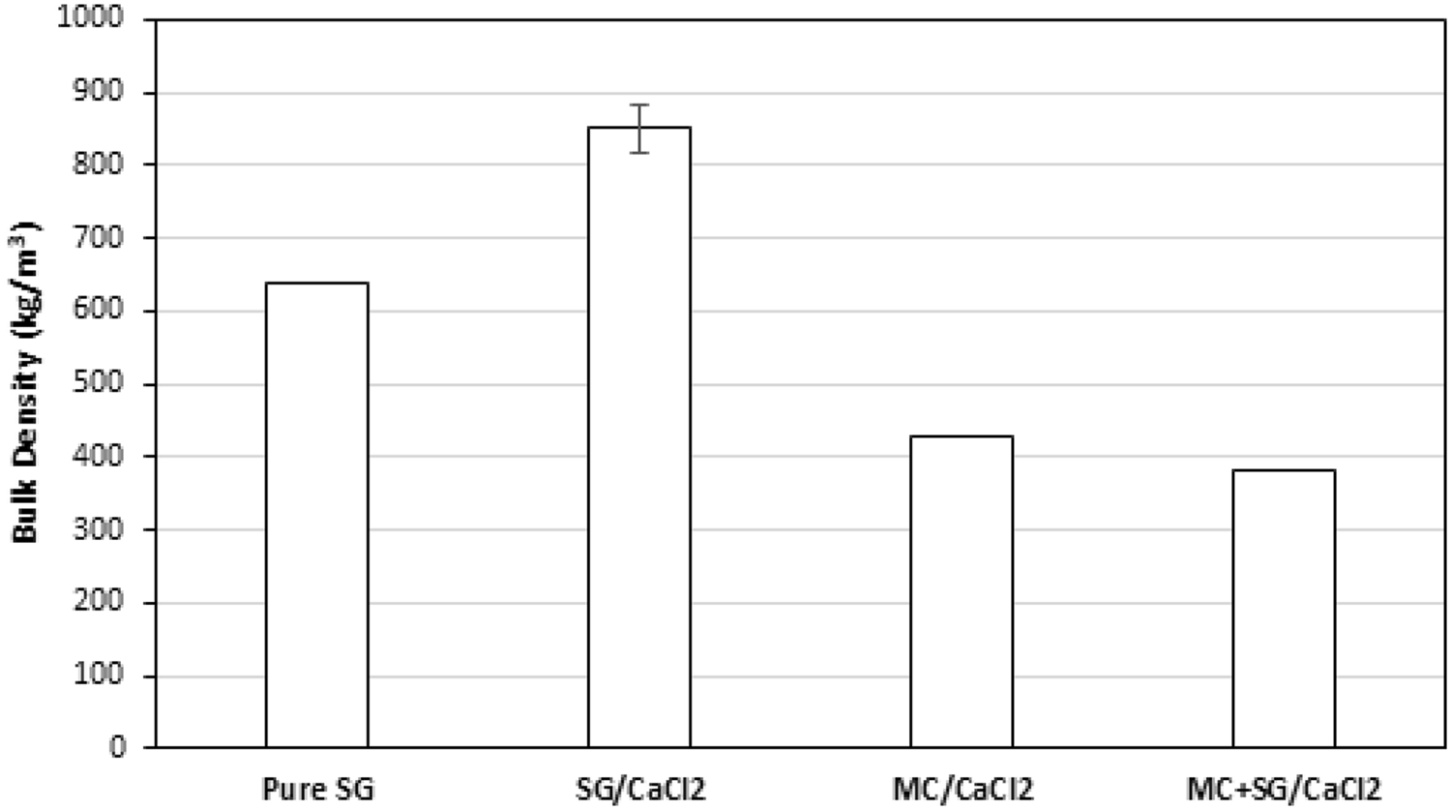
Measured bulk density of the samples used in this study. Note that the error bar for the SG/CaCl_2_ sample is based on repeated measurements for this material.

The pure silica gel showed low performance apart from a relatively high maximum thermal power, but it had excellent stability and its performance did not decrease during the three hydration/dehydration cycles. The SG/CaCl_2_ sample had high ESD and SE on the first cycle but much lower ESD and SE in subsequent cycles. This is likely due to the salt not being properly bound to the silica gel and leaving to column during hydration. Its performance after multiple cycles is getting close to that of pure silica gel, since most of the salt has left the matrix. The MC/CaCl_2_ and MC + SG/CaCl_2_ also have high ESD and SE values and showed more stability than the SG/CaCl_2_ sample for these values. Both MC containing materials show a slight change in performance after the first cycle. The MC/CaCl_2_ sample exhibited slight agglomeration after the three cycles. In Fig. 8a, it appears that for the second and third cycles, all of the composites show similar ESD values, with little change. However, the SE of the MC/CaCl_2_ and MC + SG/CaCl_2_ samples are much higher. This is because the bulk density of MC/CaCl_2_ and MC + SG/CaCl_2_ were much lower than those of the SG/CaCl_2_ sample and the pure silica gel as shown in Fig. 9. The bulk density of all the adsorbents were measured by weighing the column that was filled with dehydrated sample and dividing the sample mass by the column inner volume.

**Figure 10:**
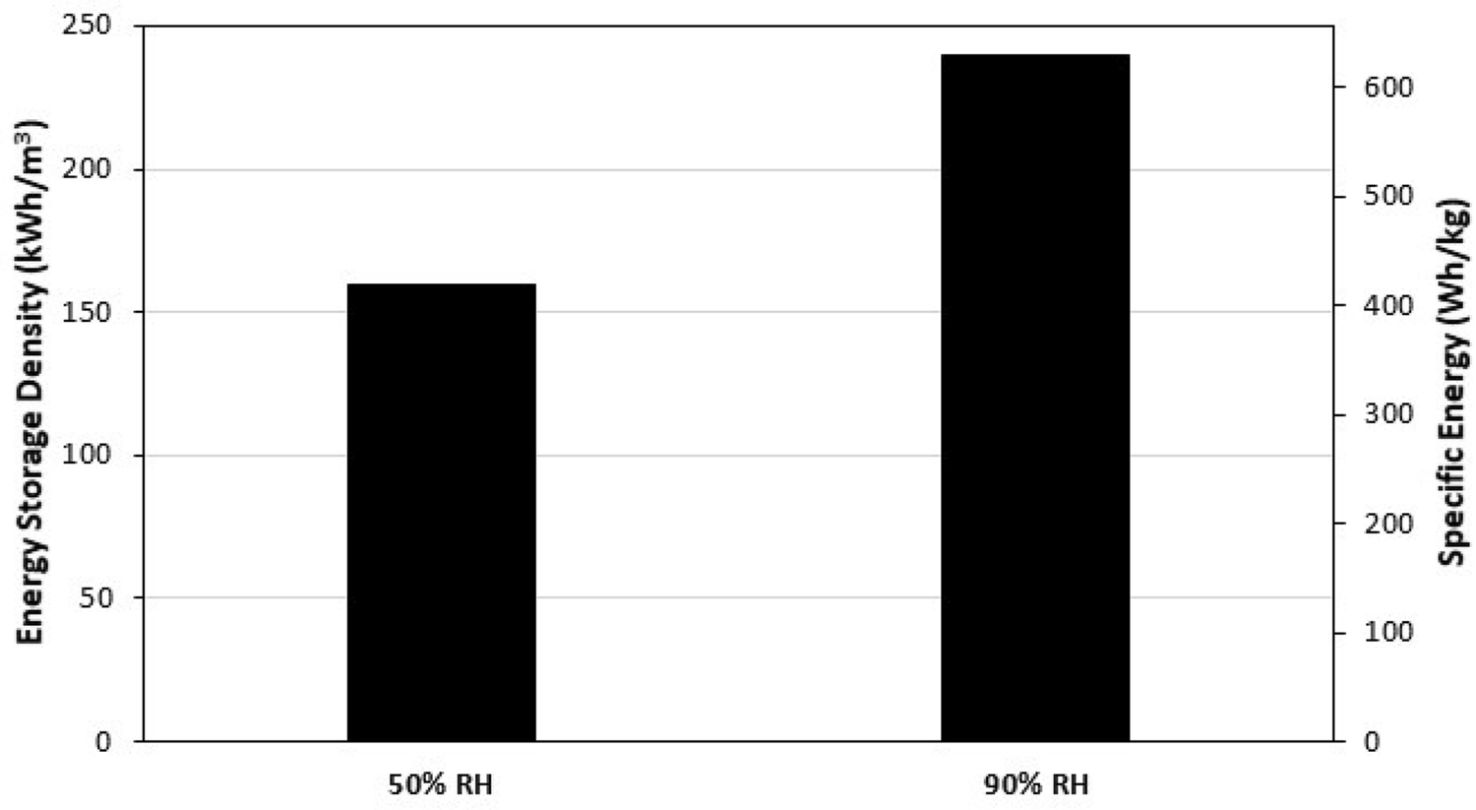
The energy storage density and the specific energy for MC/SG/CaCl_2_ at 50% inlet RH and 90% inlet RH. The regeneration temperature was 90 °C and the flow rate was 12 LPM. The value for 50% RH, is the average value of the three cycles in Fig. 8.

**Figure 11:**
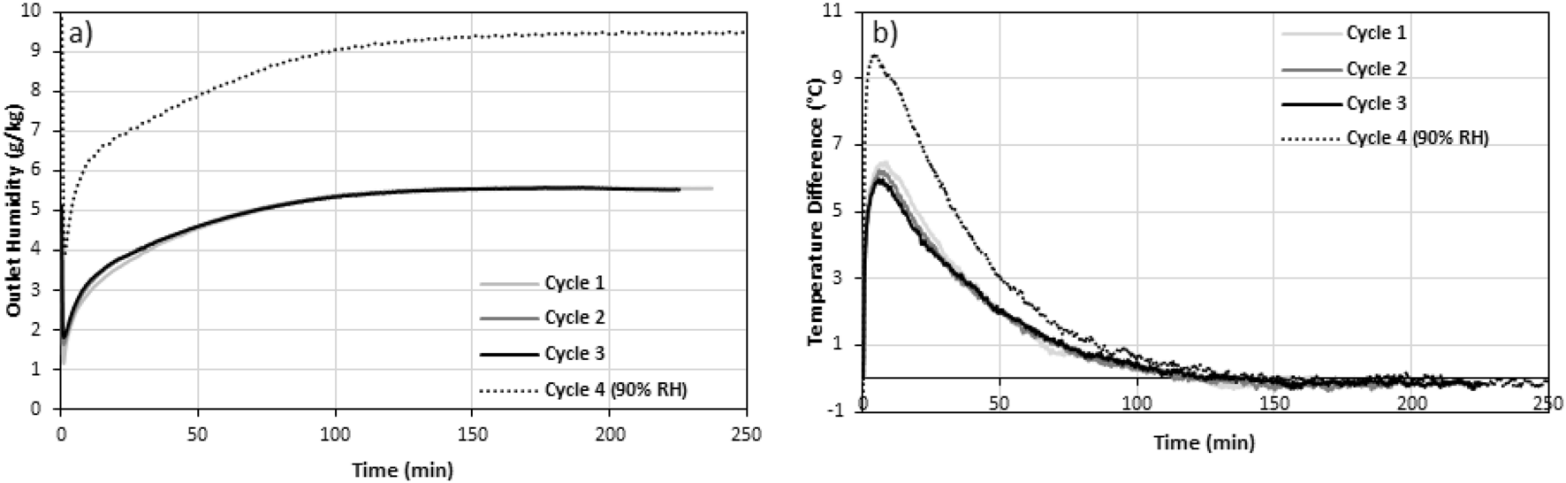
The (**a**) concentration and (**b**) temperature breakthrough curves for MC/SG/CaCl_2_ for 3 cycles (cycles 1–3) at 50% inlet RH and one cycle (cycle 4) at 90% inlet RH. The regeneration temperature was 90°C and the flow rate was 12 LPM for all experiments.

The MC + SG/CaCl_2_ sample exhibited the reasonably high energy storage performance and stability out of the tested materials, and unlike MC/CaCl_2_ it did not exhibit practical issues like swelling or agglomeration. As such, a fourth dehydration at 90 °C and fourth hydration at an inlet humidity of 90% RH was performed with this sample. The energy storage performance and breakthrough curves under these conditions are given in Figs. 10 and 11, respectively.

The ESD and SE of the MC + SG/CaCl_2_ increased by 50% when the inlet RH was increased from 50 to 90%. Additionally, the concentration and temperature breakthrough behaviours were significantly affected. The slope of the concentration breakthrough curve at the start of the experiment is much larger when the inlet RH is 90% and the maximum temperature difference is about 4 °C higher than the three cycles at an inlet RH of 50%. The reason for this increase in performance for the higher RH value is the fact that there is more moisture to be adsorbed from the air. This increases heat released during the adsorption, causing the ESD and SE values to be increased.

**Figure 12:**
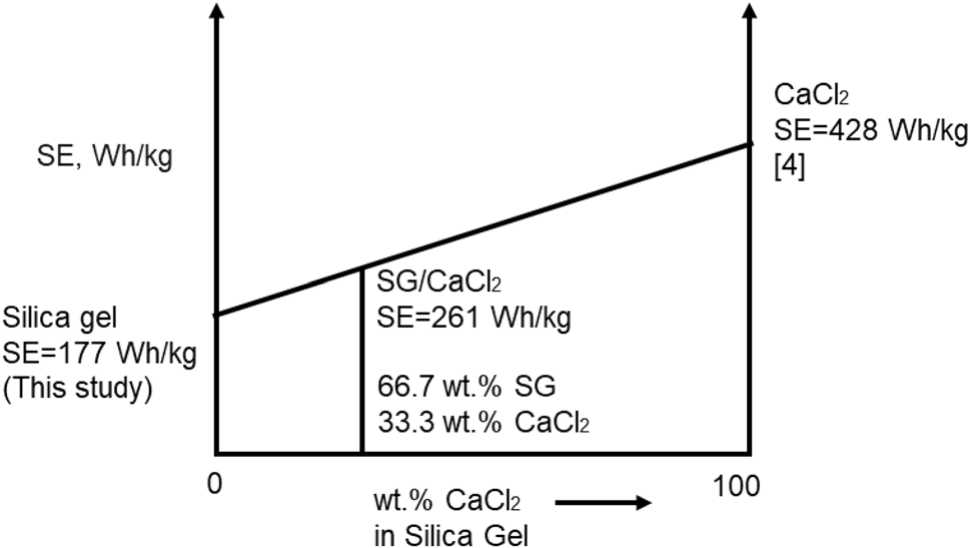
Ideal estimation of the specific energy of SG/CaCl_2_ composite.

The ideal specific energy was calculated by doing a simple specific energy estimation which is shown in Fig. 12. It is estimated that the ideal specific energy of 33.3 wt.% of CaCl_2_ and 66.7 wt.% of SG would be 261 Wh/kg. As can be seen from Fig. 8c the first cycle SE value is slightly higher than this number, but for the second and third cycles, the specific energy of the composite is much less, since CaCl_2_ leaves the column with the moisture during the hydration cycles, decreasing its amount in the adsorbent matrix.

**Figure 13:**
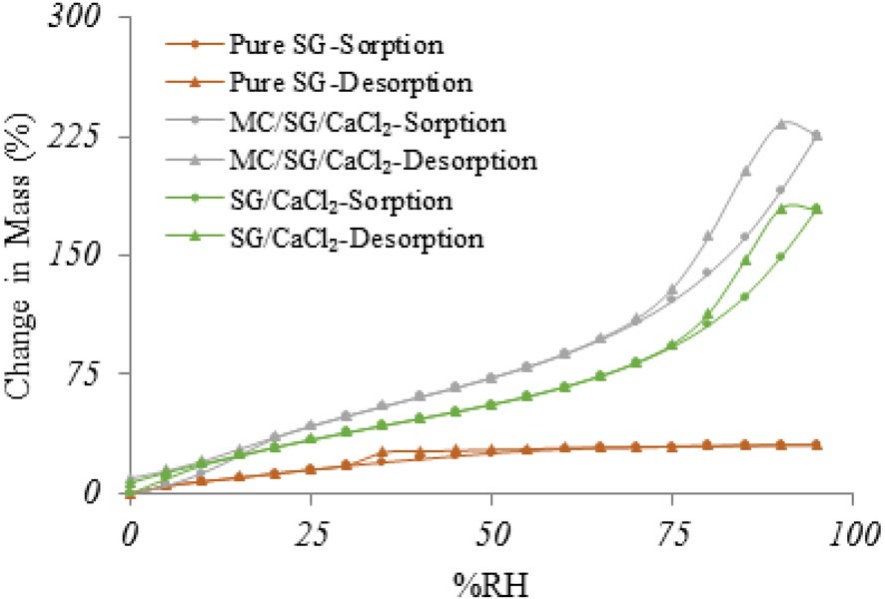
Water vapour adsorption isotherms for pure SG, SG/CaCl_2_, and MC/SG/CaCl_2_ hybrids at 25 °C.

To investigate the performance of MC/SG/CaCl_2_ hybrid further, a comparison between pure SG, and SG/CaCl_2_ has been done by measuring the water vapour adsorption isotherms at 25 °C and the results are shown in Fig. 13 for pure SG, as well as the SG/CaCl_2_, and MC/SG/CaCl_2_ hybrids. It is clear from this figure that MC/SG/CaCl_2_ has a much higher moisture adsorption capacity compared to SG/CaCl_2_ and pure SG with a capacity of 234 (w/w) at 90%RH. From this figure it can be observed how incorporation of CaCl_2_ and then MC changes the water vapour sorption performance of the SG. Pure SG is showing a Type-I isotherm and SG/CaCl_2_ and MC/SG/CaCl_2_ show the Type-II isotherm and the shape is the result of unrestricted monolayer-multilayer adsorption up to high %RH. This indicates that filling of the pores and open cavities occur at lower RH levels, whereas the capillary condensation requires higher water activity^38^. From these water vapour isotherms it is clear that the highest water vapour adsorption is for the MC/SG/CaCl_2_, which is also supporting the better performance of this hybrid compared to others. The isotherms for hybrids showed hysteresis probably due to surface adsorbed water migrating into the bulk during sorption but not during desorption^39^.

**Figure 14:**
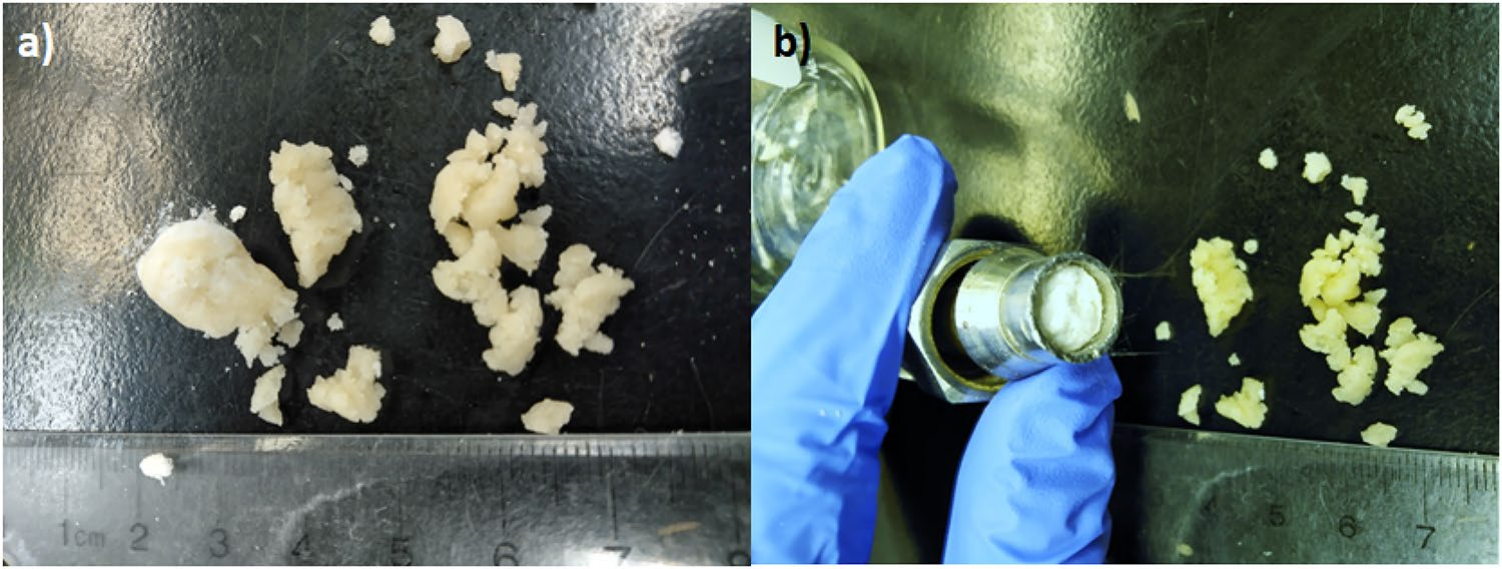
Photos of the MC/SG/LiCl composite after a dehydration at 90 °C and 12 LPM and a hydration at 50% inlet RH where (**a**) Significant particle agglomeration was observed. (**b**) A filter cake was formed on the glass wool at the exit of the column, blocking the flow through the column. Note that the original particle size was 0.841–2.83 mm before the hydration experiment.

LiCl-based composites were also synthesized and tested since LiCl has high heat of adsorption and water vapour uptake capacity^4^. However, it swells considerably more than CaCl_2_ and its deliquescence relative humidity is lower^4^. Therefore, when the LiCl samples were tested in the lab-scale energy storage apparatus, there were significant issues of deliquescence, swelling, and agglomeration (see Fig. 14a). This even resulted in a cake being formed on the glass wool at the exit of the column as shown in Fig. 14b). These issues ultimately caused an increase in pressure drop in the column as the hydration experiment progressed until the flow was completely blocked by the agglomerated particles, causing the release of a pressure relief valve. Therefore, the LiCl samples could not be practically tested using the same system as the CaCl_2_-based composites.

## Conclusions

In this study, three CaCl_2_-based composites (SG/CaCl_2_, MC/CaCl_2_, and MC/SG/CaCl_2_) were synthesized for low temperature long-term TES applications. The SG/CaCl_2_ sample was synthesized by impregnation method, the MC/CaCl_2_ composite was synthesized by encapsulation method and MC + SG/CaCl_2_ sample was synthesized by employing both the impregnation method and the encapsulation method. CaCl_2_ has high energy storage performance but needs to be stabilized as it has low deliquescence relative humidity, and it was shown that impregnation and encapsulation are effective ways to enhance performance and stability. The structural characterization of the composites was carried out by SEM and N2 isotherms at 77 K. The SEM images of the hybrids show the highly porous structure. It was observed that on incorporation of SG, porosity increases, which helps the adsorbent to adsorb more water vapour from the air carrying the moisture. All the materials prepared and the pure silica gel sample were tested using a lab-scale energy storage apparatus at 12 LPM flow rate, as well as the inlet RH value of 50%, after regeneration at 90 °C. The SG/CaCl_2_ composite exhibited high energy storage performance on the first cycle (ESD = 249 kWh/m^3^ and SE = 294 Wh/kg), but its performance decreased substantially in the second and third cycles. The MC/CaCl_2_ sample exhibited high energy storage performance (ESD = 174 kWh/m^3^ and SE = 406 Wh/kg) and high stability after 3 cycles, but it exhibited some mild swelling and agglomeration, despite the column being packed very lightly. The MC/SG/CaCl_2_ composite had high stability and energy storage performance (ESD = 156 kWh/m^3^ and SE = 407 Wh/kg), and it did not exhibit any practical use issues like the MC/CaCl_2_ composite. This implies that using impregnation and encapsulation simultaneously results in high stability composites without a sacrifice in performance.

Water vapour adsorption isotherms were measured for pure SG, as well as the SG/CaCl_2_, and MC/SG/CaCl_2_ hybrid materials. MC/SG/CaCl_2_ showed much better adsorption capacity for water vapour, confirming its better performance compared to the other two adsorbents.

LiCl-based composites were also synthesized, and an attempt was made to test them in the lab-scale energy storage apparatus. Due to issues with deliquescence and swelling, particle agglomeration occurred, and a cake was formed at the exit of the column. This caused an increase in pressure drop as the hydration progressed and ultimately blocked the flow through the column, making it impossible to test the material performance.

Since the MC/SG/CaCl_2_ composite showed high energy storage performance and stability at a hydration inlet RH of 50% for 3 cycles, a fourth cycle was performed for this sample at an inlet RH of 90%. This resulted in a 50% increase in the energy storage density and specific energy. Additionally, the maximum temperature lift increased by roughly 4 °C at a flow rate of 12 LPM.

Among the composites studied in this work, MC/SG/CaCl_2_ composite showed good stability after the 2nd cycle and good energy storage performance at 50% RH and 90% RH, at a flow rate of 12 LPM after regeneration at 90 °C, which makes this a good candidate for thermal energy storage applications. At 90% RH at the inlet, an ESD of 241 kWh/m^3^ (0.87 GJ/m^3^) and SE of 630 Wh/kg (2268 kJ/kg) were achieved with this material. Since it is known that operating conditions have a significant impact on energy storage performance of sorption-based energy storage systems^40–42^, further experiments could be performed under different conditions and the cyclic stability could be investigated further before this material is used for practical applications. Furthermore, other salts (e.g. MgSO_4_, CuCl_2_), matrices (e.g. zeolite 13X, activated alumina), and encapsulation materials (e.g. ethyl cellulose) can be used to synthesize similar composites that may also exhibit favorable thermal energy storage properties.

## Data Availability

The datasets used and/or analysed during the current study are available from the corresponding author on reasonable request.

## List of symbols

${C}_{p,air}$: Heat capacity of air (kJ/kg °C)
$H$: Absolute humidity (g water/g total)
${H}_{inlet}$: Absolute humidity at the column inlet (g water/g total)
${H}_{outlet}$: Absolute humidity at the column outlet (g water/g total)
L: Length of the column (cm)
$\dot{m}_{air}$: Mass flow rate of air (g/min)
${M}_{air}$: Molar mass of dry air (g/mol)
$M_{{{\text{H}}_{2} {\text{O}}}}$: Molar mass of water (g/mol)
$p_{{{\text{H}}_{2} {\text{O}}}}^{sat}$: Saturation vapour pressure of water (kPa)
*P*: Pressure (kPa)
*P*^*o*^: Vapour pressure of nitrogen (kPa)
${P}_{tot}$: Total pressure (kPa)
$q$: Water vapour uptake capacity (g water/g adsorbent)
${Q}_{hydration}$: Energy released during hydration (kJ)
$\dot{Q}_{\max }$: Maximum thermal power (W)
$$RH$$: Relative humidity (%)
$$t$$: Time (min)
$${T}_{in}$$: Inlet temperature (°C)
$${T}_{out}$$: Outlet temperature (°C)
V: Volume of the column (cm^3^)
$x_{{{\text{H}}_{2} {\text{O}}}}$: Mole fraction of water in the air (-)

## Greek letters

$\Delta {T}_{{\text{max}}}$: Maximum temperature difference (°C)
${\rho }_{bulk}$: Bulk density (kg/m^3^)
⌀: Diameter (cm)

## Abbreviations

BET: Brunauer–Emmett–Teller
CaCl_2_: Calcium chloride
DSC: Differential scanning calorimetry
EDX: Energy dispersive X-ray analysis
ESD: Energy storage density
LiCl: Lithium chloride
LPM: Liters per minute
MC: Methyl cellulose
MOF: Metal organic framework
PCMs: Phase change materials
RH: Relative humidity
SE: Specific energy
SEM: Scanning electron microscopy
SG: Silica gel
TES: Thermal energy storage
TGA: Thermogravimetric analysis
